# Mapping Cholesterol Interaction Sites on Serotonin Transporter through Coarse-Grained Molecular Dynamics

**DOI:** 10.1371/journal.pone.0166196

**Published:** 2016-12-01

**Authors:** Mariarosaria Ferraro, Matteo Masetti, Maurizio Recanatini, Andrea Cavalli, Giovanni Bottegoni

**Affiliations:** 1 CompuNet, Istituto Italiano di Tecnologia, Genova, Italy; 2 Department of Pharmacy and Biotechnology (FaBiT), Alma Mater Studiorum - Università di Bologna, Bologna, Italy; 3 BiKi Technologies s.r.l., Genova, Italy; Consiglio Nazionale delle Ricerche, ITALY

## Abstract

Serotonin transporter (SERT) modulates serotonergic signaling via re-uptake of serotonin in pre-synaptic cells. The inclusion in cholesterol-enriched membrane domains is crucial for SERT activity, suggesting a cross-talk between the protein and the sterol. Here, we develop a protocol to identify potential cholesterol interaction sites coupling statistical analysis to multi-microsecond coarse-grained molecular dynamics simulations of SERT in a previously validated raft-like membrane model. Six putative sites were found, including a putative CRAC motif on TM4 and a CARC motif on TM10. Among them, four hot-spots near regions related to ion binding, transport, and inhibition were detected. Our results encourage prospective studies to unravel mechanistic features of the transporter and related drug discovery implications.

## Introduction

Serotonin transporter (SERT) is a membrane protein of the neurotransmitter sodium symporters (NSS) family. These secondary active transporters couple the diffusion of sodium, chloride and potassium ions down their electrochemical gradients to drive the re-uptake of substrates against their concentration. Mediating the translocation of serotonin into pre-synaptic cells, SERT represents a key determinant in the regulation of serotonergic signaling and, as such, a relevant pharmaceutical target for the treatment of depression and drug addiction [1]. Several NSSs [2], including SERT [3], are associated to cholesterol-enriched membrane domains, called lipid rafts, which concur to transport modulation through changes in membrane properties or direct binding to specific sites on the protein surface [4–7]. Since transport rates are decreased in cholesterol depleted membranes in a dose dependent manner [8], it has been recently proposed that this sterol might regulate SERT functionality by inducing a transient conformation with high affinity for serotonin (outward-open state) [9]. Similar considerations hold for the closely related Dopamine transporter (DAT), whose conformational equilibrium is shifted toward the outward-open state in raft-like membranes [10]. However, a direct evidence for NSS-cholesterol interactions has been only recently provided with the solution of several eukaryotic DAT crystal structures [11–13]. Indeed, all the reported *d*DAT (*Drosophila Melanogaster*) crystals show one or two cholesterol molecules bound in transmembrane (TM) crevices of the transporter: the first is a cleft between helices TM1, TM5, and TM7, while the second is located in proximity of TM2, TM7, and TM11. Notably, these regions are involved in substrate translocation and are mostly conserved among NSS homologues proteins [14]. The growing evidence for a cross-talk between proteins and lipids has called in recent years for an in-depth investigation of these regulatory mechanisms at the experimental and theoretical level [15–17]. In spite of this, substantial progresses in computational modeling have long been hampered by the lack of human form crystals. A major breakthrough in the field has come with the deposition in the Protein Data Bank of the first high-resolution (1.65 Å) structure of a bacterial member of the NSS family, the Leucine transporter (LeuT, pdb ID: 2A65) [18]. This occurrence, combined with the release of the first eukaryotic DAT crystal (*d*DAT, pdb ID: 4M48) [11], have opened the way to the development of several homology models of human NSS homologues [19]. In particular, these two orthologues proteins show a remarkable similarity in their tertiary structure, especially in the membrane spanning helices, which are organized in a pseudo two-fold inverted symmetry relating TM1-5 to TM6-10. Conversely, the loop regions, the N- and C-termini, and the peripheral TM11-12 helices reside out of the symmetric motif and, being the less conserved structural portion, are also responsible for intra-species selectivity among closely related NSSs [11, 18]. Although such differences contribute to a low overall sequence identity (~ 20–30%), as revealed by molecular dynamics (MD) simulations and inferred by several crystal structures [20, 21], these proteins share a common transport pattern, whereby the transition from the outward-open to the inward-open state is achieved through a rocking-bundle mechanism [22]. The suitability of LeuT in describing NSSs pharmacology has been recently addressed and thoroughly reviewed in the light of the latest eukaryotic structures released [19, 23, 24]. Moreover, the very good agreement between ligand binding modes in NSSs built from LeuT and *d*DAT, underlines the actual possibility to generate effective predictive models [19, 25–28]. In this respect, recent studies have been carried out on both LeuT- and *d*DAT-based homology models to investigate the effects of *h*DAT missense mutations on the onset of brain diseases [29]. A similar conclusion can be drawn the investigation of NSSs-lipid interactions, where the above mentioned homology models have found broad application [30–32].

Here, we present multi-microsecond coarse-grained molecular dynamics simulations (CG MD) of a state-of-the-art *h*SERT homology model in a raft-like membrane environment validated in our recent work [33]. Combining two complementary analysis approaches, we developed and applied a systematic protocol to identify potential cholesterol interaction sites in regions crucial for ion binding, translocation and inhibition. Two of them represent CRAC/CARC motifs [34], which are membrane protein sequences known to interact with sterol molecules. Moreover, our simulations successfully identified a hot-spot that overlaps with the cholesterol binding site in *d*DAT crystal structure (pdb ID: 4M48) [11], that is overall conserved in *h*SERT.

To the best of our knowledge, our study is the first computational report on the interaction pattern between cholesterol and a member of the NSS family simulated in a raft-like membrane model. The main goal of the present work is to identify SERT-cholesterol interaction sites, providing structural basis to support the direct and specific binding between the two species [3, 8, 9]. Our results supported by a strong theoretical framework encourage further in-depth analyses aimed at unraveling mechanistic features of such cholesterol-mediated modulation. Moreover, we developed a systematic method which can be broadly applied to different biological systems or functional lipid species. Finally, unveiling new specific mechanisms in SERT could hopefully lead to discover novel druggable allosteric regions on this important drugs target.

## Models and Methods

### *h*SERT homology model

In this study we used an experimentally validated *h*SERT homology model developed and made accessible by Celik and co-workers [26]. The model was built using the outward-occluded state of LeuT (PDB id: 2A65) as a template [18], and then refined through docking, SAR and mutagenesis studies within the framework of Paired Mutant-Ligand Complementation (PaMLAC) paradigm [25, 26]. RMSD deviations smaller than 2 Å in the TM regions of inference substantiate the use of LeuT as a suitable template for NSSs [29, 35]. In this way, it was possible to obtain a state-of-the-art model retrospectively validated by the recently published eukaryotic crystal structures.[36] Relying on the quality of the provided model and on studies showing that cholesterol modulates DAT and SERT outward facing conformations [9, 10], we reasoned that the transporter in an outward-occluded state could be a suitable starting point to investigate cholesterol binding. Indeed, in the occluded state, the transporter is still in an outward conformation, although small TM rotations in proximity of the substrate binding site are expected [13].

Since there is clear evidence that *h*SERT is present at membrane surfaces as covalent dimers, tetramers, and higher-order oligomers, the dimeric form was considered in our simulations [37, 38]. An advantage of using a dimeric transporter is the possibility to double the statistics collected on each system through the use of a single simulation box. The dimeric structure has been successfully used in previous works.[20, 39–41]. For further justifications on the use of LeuT as template and the chosen dimeric assembly (Fig A in S1 Supporting Information).

### Model systems

To perform our simulations, we used the MARTINI force field in the 2.2 version for protein residues [42] and 2.0 for lipids [43], as implemented in Gromacs v 4.6 [44].

A reliable lipid environment was mimicked taking advantage of our previously validated membrane models containing 15, 20 and 25 mol% of cholesterol with phosphocoline (PC) and phosphoethanolamine (PE) derivatives [33]. Increasing molar ratios were considered to investigate potential effects on cholesterol binding and on the duration of interactions. Each mixture was assembled with a homo-dimeric form of SERT, through a self-assembly procedure (see S1 Supporting Information, section 2.1), so that every system experienced a different starting configuration of membrane lipids. The three resulting systems are named as SYS1-3 according to increasing cholesterol concentration. Each system was assembled and then simulated in at least two independent copies, labeled as SYSX.1 and SYSX.2, for 30 μs. This corresponds to a total of 360 μs monomer-based trajectories. To avoid any memory of the initial state we discarded the first 500 ns from each run. Since two SERT monomers per system were available, these simulations resulted into twelve individual data-sets, hereafter named as SYSX.Ya/b, whose statistical significance and population membership were carefully addressed as described in S1 Supporting Information (Section 2.2). Moreover, to assess the sampling exhaustiveness with the chosen amount of data, a third copy of SYS3 was added (SYS3.3) and simulated for further 30 μs (bringing to fourteen the number of available data-sets) (see S1 Supporting Information, section 2.3).

### Analysis of trajectories: Protocol outline

In order to map the cholesterol interaction sites on the surface of the SERT model, a distance cutoff of 6 Å was used to define a contact between all the possible bead-pairs of the sterol and protein residues. This value has been already employed in similar studies, and approximates the first minimum in the radial distribution function for MARTINI models [45, 46]. The maximum occupancy time (*t*_max_) [47], which is the longest time a residue contacts a single cholesterol molecule, was used to detect specific interaction sites. This variable discriminates between random, short-lived events, and specific, long-lasting ones. While these latter are characterized by microsecond long *t*_max_, the former typically occur on a nanosecond timescale [47]. Starting from the collected *t*_max_ values, we apply the procedure detailed in the Expanded Methods section of S1 Supporting Information, which is briefly summarized as follows:

1. Statistical analysis of *t*_max_ distributions to identify the presence of outliers (residue-based analysis). Since specific interactions are associated to exchange rates of the order of microseconds, outliers showing *t*_max_ values significantly displaced from the central tendency, represent anomalous data worthy of further investigation. Moreover, to rationalize the effect of cholesterol on the interaction patterns, the information from all systems was combined through careful statistical analysis, resulting in a single set of outliers.
2. Calculation of the spatial density probability function (SDF) for all cholesterol beads around each SERT monomer. In the limit of exhaustive sampling, residues with high *t*_max_ are expected to match sites with high cholesterol density [47]. When possible, trajectories belonging to a SYSX were merged following information derived from a statistical test of hypothesis. SDF was calculated on such merged populations (or macro-samples), resulting in three unique density maps, to reveal only entirely reproducible and spatially defined hot-spots. Reproducibility among different systems and cholesterol stability were judged as important criteria to define the topology of the sites.
3. Superimposition of the SDF hot-spots to residues corresponding to the identified outliers. The agreement achieved between high cholesterol densities and statistically significant *t*_max_ in each interaction site was taken as a proxy of biological relevance and used as a ranking parameter.
4. Site-based analysis to characterize cholesterol’s residence times. The analysis was performed only for SYS3, which more closely mimics sterol enrichments in “atypical” rafts [33, 48].

In addition, we also addressed the selectivity of the identified sites. To this aim, the maximum occupancy times for the other lipid components (PC and PE) were extracted for SYS3, averaged over the individual data-sets, and compared with cholesterol *t*_max_.

#### Cholesterol’s spatial density probability function

To capture the existence of preferred sites of interaction around SERT surface, spatial density probability function (SDF) for all cholesterol beads was computed. To take into account the increased size of MARTINI beads, the minimum volume element was set to 1 Å^3^. The SDF is defined as:

$$g\left(x,y,z\right)=\frac{N\left(x,y,z\right)}{\rho \times \Delta V\times N_{frames}}$$ (1)

where *N*(*x*,*y*,*z*) is the number of atoms counted in a bin during the simulation, which is divided by the volume of a bin (Δ*V*), the number of frames (*N*) and the reference density (*ρ*). The latter is obtained dividing the number of atoms by the total volume of the box. Trajectories collected on monomers belonging to the same macro-sample (see S1 Supporting information, section 2.2), were fitted on the same 466 SERT membrane-facing residues employed for the *t*_max_ analysis, and merged consistently. The SDF was calculated for SYS1, SYS2 and SYS3 through the tool *g_spatial* available in Gromacs 4.6.

#### Site-based characterization of cholesterol’s residence times

By mapping statistical outliers and reproducible cholesterol spots onto the SERT surface, we were able to appreciate the agreement between the results of the two analyses in specific areas of the transporter, so as to allow a topological characterization of the sites.

After least-squares fitting of monomeric trajectories belonging to SYS3 on the 466 analyzed residues, cholesterols contacting the SERT’s surface over 118 μs were identified by the previously introduced distance-based cutoff (6 Å). Then, single trajectories for the identified lipid molecules were extracted and analyzed through the tool *g_rms* available in Gromacs 4.6. Cholesterol binding dynamics within the sites was analyzed mapping each molecule on its reference bound pose, mirroring SDF surface, and using a double RMSD cutoff to describe binding and unbinding events. In particular, a binding event was associated to an RMSD lower than 6 Å, whereas values higher than 10.8 Å were chosen to describe a completely unbound state. The introduced tolerance helped us to avoid the overestimation of the unbound state, due to plastic binding and partial unbinding which is associated to cholesterol interaction sites [47]. Similar RMSD threshold has been previously used in an analogous study [49]. Through these definitions we obtained the time spent by cholesterol in each site,that is hereafter referred to as “residence time” to be distinguished by the residue-based definition of maximum occupancy time (*t*_max_, see S1 Supporting Information, Section 2.2).

## Results and Discussion

### Distribution of maximum occupancy times

**Fig 1:**
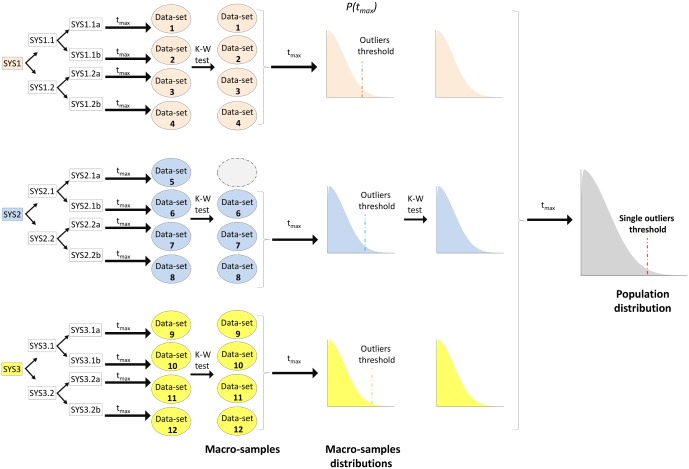
Schematic picture of data processing and statistical analysis carried out on SYS1-3. Data-sets, macro-samples and population distributions are shown and colored accordingly to each system (K-W: Kruskall-Wallis). For sake of clarity, the assessment of sampling convergence involving a third copy of SYS3 (SYS3.3) is not explicitly shown.

In Table 1 we report the maximum occupancy times calculated for the twelve individual data-sets (plus the additional sets for SYS3, see section 2.3 in S1 Supporting Information for details) and the corresponding statistical descriptors. The D’Agostino-Pearson normality test returned p-values << α = 0.01, confirming that the distributions were not normal (Table A in S1 Supporting Information). The Kruskall-Wallis test provided values of 1.49, 20.01 and 6.15, for the four data-sets of SYS1, SYS2 and SYS3, respectively. At the 0.01 level of significance, SYS1 and SYS3 (H < χ^2^ = 11.34) successfully passed the test and data obtained from the four respective monomers were merged to obtain a unique distribution. On the contrary, the test rejected the null hypothesis that the four samples of SYS2 came from the same populations, indicating some statistical divergence of their medians. In order to identify the outlier trajectory in SYS2, we repeated the Kruskall-Wallis test iteratively leaving each one of the monomer out from the analysis. The resulting H values are reported in Table B in S1 Supporting Information. At a 0.01 level of significance for two degrees of freedom, H must be lower than the tabulated χ^2^ of 9.21. Using this strategy, we found that the null hypothesis was not rejected for two out of four possible combinations of triplets. Relying on statistical definition of Kruskall-Wallis test we chose to merge trajectories belonging to the three data-sets: SYS2.1b-SYS2.2(a/b) (Fig 1). By discarding SYS2.1a, the total number of data-sets was reduced from twelve to eleven.

**Table 1:** Data spread of t_max_ distributions obtained from all monomers within SYS1-3, including SYS3.3.

System	Data-sets	Median (ns)	No. of contacting residues	1^st^ quartile (ns)	3^rd^ quartile (ns)	Outlier threshold *T* (μs)
SYS 1	1a	33.1	320	4.5	113.6	0.277
	1b	36.0	326	4.0	116.1	0.284
	2a	45.1	298	4.4	152.3	0.374
	2b	38.0	305	4.2	133.2	0.327
SYS 2	1a	30.0	324	2.9	120.1	0.296
	1b	62.0	324	7.0	229.0	0.562
	2a	52.5	324	6.0	180.6	0.443
	2b	42.0	315	3.6	164.6	0.406
SYS 3	1a	68.3	312	6.7	277.3	0.683
	1b	61.6	306	7.6	238.7	0.585
	2a	64.0	327	6.4	200.6	0.492
	2b	42.5	328	4.5	180.4	0.444
	3a	65.0	319	7.8	243.2	0.596
	3b	54.6	329	5.9	199.7	0.490

Once maximum occupancy times were merged, three unique distributions were obtained for SYS1, SYS2 and SYS3 (Table B and Fig B in S1 Supporting Information) and successfully passed the previously described test for normality (see p-values in Table A in S1 Supporting Information).

By comparing the statistical descriptors of *t*_max_ distributions obtained from individual data-sets and from the corresponding macro-samples, some variability emerged among the medians (Table 1), with the most striking difference being in data-sets belonging to SYS1, in which central tendencies were substantially lower than those in SYS3. However, merging four or three data-sets, a better agreement among populations was observed (Table 2). The Kruskall-Wallis test confirmed that, at a 0.01 level of significance there were no statistical differences in the medians of the three macro-samples (H = 4.93 < χ^2^ = 9.21; df = 2). While the cholesterol enrichment in SYS3 increases the probability of interaction with SERT compared to SYS1-2, the rate of cholesterol diffusion actually prevented this achievement. In fact, the diffusion coefficient of the sterol acts as a rate limiting step for the formation of lipid-protein complexes [50], and it decreases going from SYS1 to SYS3 [33]. Collecting and merging adequate statistics, we found that the faster lipid diffusive behavior in SYS1 can compensate for the reduced number of interacting molecules in the system.

**Table 2:** Data spread of t_max_ distributions obtained merging single data-sets and macro-samples as described by statistical test of hypothesis (Kruskall-Wallis).

Samples	Length of aggregated trajectories (μs)	No. of contacting residues	Median (ns)	1^st^ quartile (ns)	3^rd^ quartile (ns)	Outlier threshold *T* (μs)
SYS1	118.0	361	67.0	7.4	229.1	0.6
SYS2	88.5	356	80.6	7.0	320.0	0.8
SYS3	118.0	356	96.6	8.9	406.8	1.0
SYS 1-2-3	324.5	389	125.2	11.3	531.5	1.3

A considerable advantage of working with merged samples distribution was that statistical descriptors of the corresponding populations were more representative of the events of interest. Indeed, population median of the least concentrated system (Table 2) was observed to approach medians obtained for individual samples in SYS3 (Table 1). In light of these results, we showed that with substantial sampling and properly merged distributions, the effects of decreased diffusion rates on the underlying population of *t*_max_ were damped and in general overcome. This was also supported by constant numbers of contacting residues and statistical evidence from Kruskall-Wallis test. For this reason, we then focused on SYS3 by assessing whether the highest cholesterol concentration could significantly enhance the probability to sample additional molecular events in a triplicate independent simulation (SYS3.3). Statistical descriptors for the two additional data-sets can be found in Table 1 and Table A in S1 Supporting Information. The resulting distributions obtained with six monomers passed the Kruskall-Wallis test (H = 7.77 < χ^2^ = 15.08; α = 0.01; df = 5) for the equality of population medians, indicating homogeneous sampling among monomers. Comparing *t*_max_ distributions obtained from SYS3.1–2 and SYS3.1-2-3, via Wilcoxon Rank Sum test (see S1 Supporting Information, section 3.2), we verified the null hypothesis that no significant displacement of populations location was observed (p-value = 0.71 >> 0.01). This means that the addition of two further data-sets to the four sets originally considered did not generate a statistically relevant improvement in the global sampling of the most concentrated system. Likewise, data collected on SYS3.3 were not sufficient to diversify the three macro samples (H = 2.01 < χ^2^ = 9.21; α = 0.01; df = 2). This justified the use of two copies for each system as a starting point to identify representative outliers and draw our conclusions.

**Fig 2:**
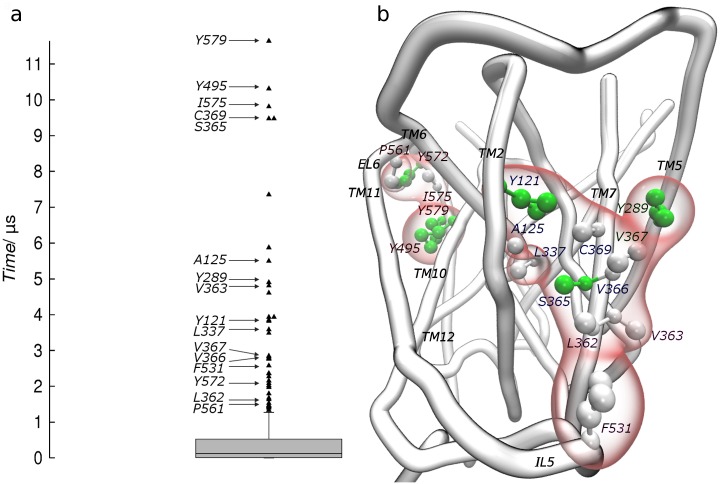
Outliers characterization. (**a**) Distribution of *t*_max_ obtained merging eleven data-sets from SYS1-3 in a unique population. The box-plot diagram shows the outliers located above 1.3 μs (black triangles). (**b**) Corresponding amino acids are only labeled for the sixteen outliers matching a reproducible cholesterol density, and are mapped on the three-dimensional representation of the SERT surface. Outliers are colored by residue type and contoured by a red surface. Labels follow the site-based coloring scheme shown in Fig 3.

Since no evidence for statistical differences emerged between the populations of SYS1-3, outlier residues were extracted from a unique *t*_max_ distribution profile (see Fig 1) and are shown in Fig 2a.

As expected, the first half of the residues was characterized by *t*_max_ in the range of nanoseconds, and a few of them were found in the outliers’ region. This result confirmed the presence of residues involved in long lasting contacts which were significantly different from the rest of the population. Little changes were observed in the number of contacting residues among systems (Tables 1 and 2), indicating that 15 mol% was sufficient to saturate all the potential interaction sites. Although we got evidence that different cholesterol enrichments did not significantly affect *t*_max_ of statistical outliers, we noticed an overall increase in maximum occupancy times in the middle portion of samples distributions going from SYS1 to SYS3 (Table 1). Indeed, the first half of samples in SYS3, describing SERT-cholesterol aspecific contacts, showed higher *t*_max_ (medians) compared to the others, probably due to a progressive decrease in membrane diffusion coefficients [33]. Notably, the time threshold for the identification of outliers’ region was 1.31 μs, in very good agreement with the microsecond time-scale associated to specific protein-cholesterol interactions [47]. These results suggest a dual behavior on SERT-cholesterol interactions: while specific contacts are only marginally affected by the cholesterol enrichment, changes in membrane dynamic properties turned out to have a substantial effect on the duration of aspecific interactions. This implies the presence of preferred thermodynamic basins on the SERT surface that will be discussed in the next paragraph.

### Characterization of the interaction sites and mechanistic implications

**Fig 3:**
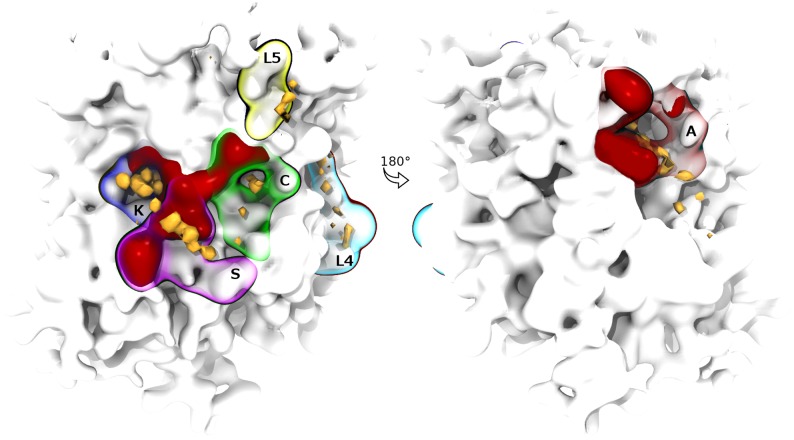
Overview of the cholesterol interaction sites mapped on the SERT’s transmembrane surface. The sites are drawn as differently colored contoured surfaces, while corresponding outliers are shown in red. Cholesterol density is normalized to the average bulk value (isovalue of 1) and displayed as yellow surfaces at a contour level of 60 (left) and 40 (right) hits/Å^3^. See Fig 2 for outliers’ identity.

**Fig 4:**
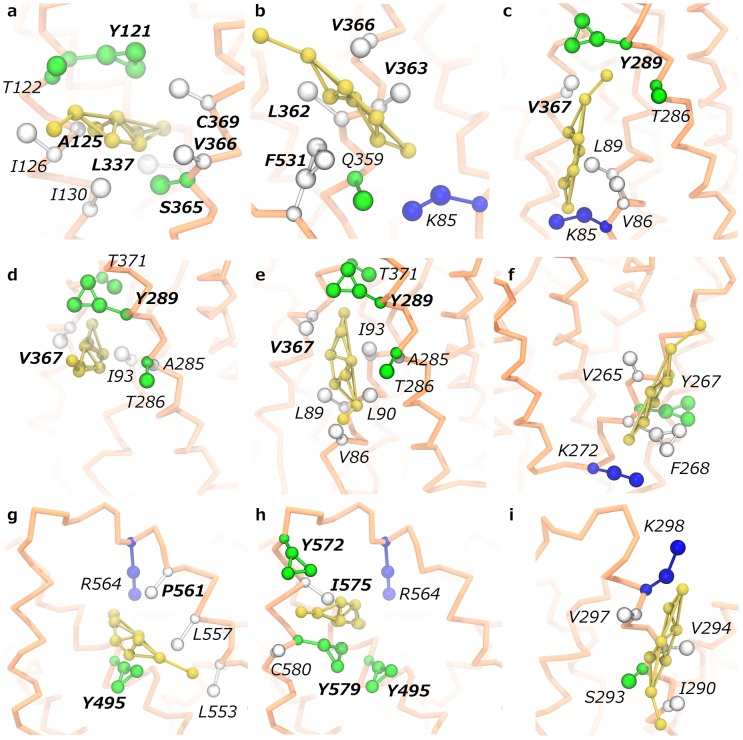
Focus on cholesterol interaction sites. **(a-i)** Coarse-grained representation of SERT residues (CPK representation; coloring scheme: apolar/aromatic: white; polar/aromatic: green; blue: basic) forming the six sites and interacting with a cholesterol molecule (yellow CPK representation) in all the observed binding modes (see text) are shown. (**a**): Site K; (**b**): Site S; (**c**): Site C, binding mode 1; (**d**): Site C, binding mode 2; (**e**): Site C, binding mode 3; (**f**): Site L4; (**g**): site A, sub-site A1; (**h**): Site A, sub-site A2; (**i**): Site A, sub-site A2. The sixteen outlier residues are labeled in bold.

By matching statistical outliers to reproducible cholesterol densities, we identified the hot-spots with a clear binding preference on the SERT surface. Through this procedure, only 16 outliers out of the 41 obtained (n = 389) via the previously described statistical analysis were retained (Fig 2b). As a result, we disclosed six potential interaction sites ranked based on the agreement between the two analyses: K, S, A, C, L5 and L4 (Figs 3 and 4).

Sites K, S, A and C are non-linear (three-dimensional) sites involving TM1, TM2, TM5, TM7, and TM10-12, whereas L sites are linear, namely sites simply defined by the protein sequence, found on TM5 (L5) and TM4 (L4) (Fig 4). K and S are well-defined sites, characterized by a dominant cholesterol binding mode preserved for several microseconds which resulted in unique and spatially confined density spots, flanked by 6 (K) and 4 (S) outliers (Fig 4a and 4b). Conversely, cholesterol was observed to interact with sites A and C in two and three preferred orientations, respectively, which were responsible for splitting both densities and *t*_max_ (Fig 4c, 4d, 4e, 4g and 4h). Here, 5 (A) and 2 (C) outliers were found in each site. Finally, L5 and L4 returned consistent SDFs, but no overlapping outliers were found (Fig 4f and 4i) (see section 5. in S1 Supporting Information, for discussion).

Once the topology of the six hot-spots was defined, we performed a comparative site-based analysis for SYS3 along the 118 μs of CG-MD (Table 3). The highest *t*_max_ obtained for single residues (~ 11 μs) was one order of magnitude lower than the total simulation time, in accord with reported time-scales of protein-cholesterol interactions [47]. We found that the number of binding events correlated to site exposure. As expected, L5 and L4 showed similar features, being the most contacted ones, with only two events beyond the microsecond. Linear sites associated to specific cholesterol binding are defined as CRAC (Cholesterol Recognition Amino acid Consensus) or CARC (if oriented in the opposite direction) motifs [34], whose function is still controversial. Indeed, site L4 was found to fulfill the requirements of a CRAC sequence (see S1 Supporting Information, section 5., for discussion). Despite the conserved signature in primary sequence (site L4), these sites have never been described in SERT.

**Table 3:** Structural and dynamic features of sites. Outlier residues conserved among *h*SERT, *h*DAT, and *h*NET are shown in bold.

*Sites*	*K*	*S*	*A*	*C*	*L4*	*L5*
Topology	Non-linear	Non-linear	Non-linear	Non-linear	Linear	Linear
No. binding modes	1	1	2	3	1	1
Residues	**Y121**, T122, **A125**, I126, I130, **L337**, **S365**, **V366**, **C369**	K85, Q359, **L362**, **V363**, **V366**, **F531**	**Y495**, L553, L557, **P561**, R564, **Y572, I575**, I576, **Y579**	K85, V86, L89, L90, I93, A285, T286, **Y289**, **V367**, T371	V265, Y267, F268, K272	I290, S293, V294, V297, K298
Conserved residues (*h*SERT, *h*DAT, *h*NET)	N368, S372, **Y121**, S336, **L337**	K85	-	L89, T286, **Y289**, A285, L90, T371	F268, Y267, K272	-
Maximum residence time (μs)	22.3	6.5	11.6	8.8	1.5	1.3
No. events (total/> 1 μs)	19/5	777/21	297/13	169/8	817/2	1106/2
Aver. No. visiting cholesterol	5	92	56	54	116	95

Non-linear A and C sites showed to bind cholesterol in different modes and experienced similar residence times, which were considerably higher than those in linear sites. In contrast, top-ranked sites K and S, while similar, behaved very differently from a kinetic point of view. Site K is located in a buried pocket between two helices and is characterized by low accessibility. This site was visited on average by only 5 molecules in each simulation, but was associated to the SDF maximum (most favored thermodynamic basin). The low number of events and high residence times suggest that cholesterol could be barely displaced by competition from such a buried site, consistently with high energy barriers for (un)binding and long-lasting contacts.

Conversely, site S, in spite of its three-dimensional topology defined by three helices and an intra-cellular loop, was characterized by substantial lipid exposure. This feature emerged from the number of events, which was an order of magnitude higher than the previous case (777 vs. 19) and nearly comparable with those found in linear sites. Yet, site S was characterized by the highest number of specific events (above the microsecond). The reason for the high occurrence of specific interactions in such region could reside in a peculiar combination of activation barriers for cholesterol (un)binding that are lower compared to site K, and at the same time more favorable enthalpies relatively to mono-dimensional sites.

Such balance between kinetic and thermodynamic properties confers to site S an intermediate binding behavior between linear and non-linear sites.

What are then the possible functional implications of cholesterol hot-spots on SERT surface? Here, we focus on non-linear sites, while the remaining ones are discussed in S1 Supporting Information (Section 5.).

**Fig 5:**
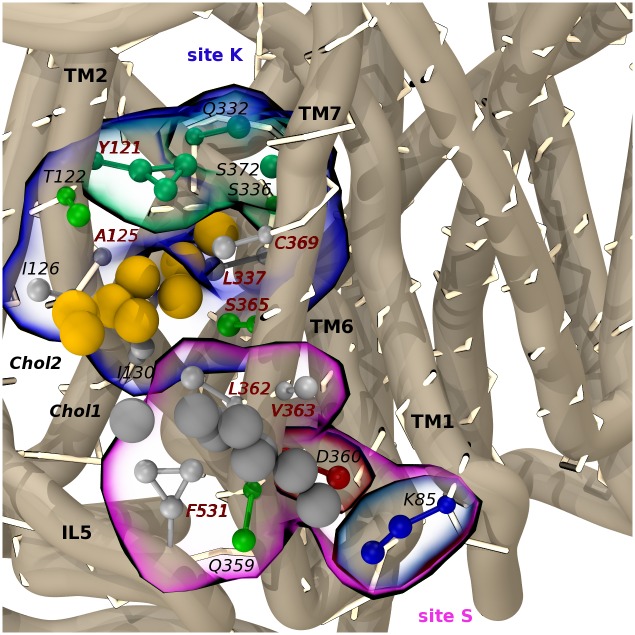
Sites S and K. Site S (purple contoured surface) and site K (blue contoured surface) bound with two distinct cholesterol molecules (Chol1 and Chol2, grey and yellow beads, respectively). Residues of chloride site (darker green CPK representation) are contoured in green transparent surface; N368 on TM7 faces the interior of the transporter and is not labeled in the picture. Outliers are labeled in bold (dark-red color). Amino acids entering the topology of the sites are colored by residue type. The K85-D360 salt-bridge is also shown.

As previously mentioned, site K is located in a buried pocket between TM2, TM6 and TM7 (Figs 4a and 5).

This region is highly conserved in SERT, DAT, and norepinephrine (NET) transporters, as it contains residues forming the chloride site in NSSs [51]. Chloride binding has been proposed to be crucial for SERT activation. Without a negative charge, key residue Y121 reorients its side-chain, resulting in a binding- and transport-incompetent SERT conformation [51]. In Fig 5, a configuration showing a cholesterol molecule in close proximity to four out of five residues composing the chloride site is reported. Favorable interactions between Y121 and sterol rings were found to influence the orientation of the amino acid’s side chain, suggesting a role for this lipid in the rearrangement of the site. Moreover, outlier A125 is not conserved in DAT and NET, where it is replaced by M106 and L102, respectively (Fig C in S1 Supporting Information). We speculate that the presence of sterically hindering residues could prevent the binding of cholesterol, making K a potential selective site for SERT modulation. Interestingly, in the recently published crystal structures of *d*DAT [13], a second cholesterol interaction site has been found in close proximity of site K. Likewise, this site involves helices TM2, TM7 and IL5, however, the lipid is not buried in the cavity between helices, rather lying at the entrance of the site and supporting our hypothesis of selectivity. In addition, we observe that TM2 and TM7 are also involved in the transition between outward- and inward-open states. According to the bacterial crystal structures of the homologous LeuT, both these helices have to bend at their midsection during the conformational change [21]. Thus, in that position, cholesterol could prevent helices bending, with the result of stabilizing the outward-open state. This is in agreement with a recent report showing that cholesterol induces an outward-open conformation in SERT with higher affinity for serotonin [9]. By slowing down the achievement of the inward-open state, however, the outward-open conformation behaves as a rate limiting step for the substrate translocation. This mechanism is proper to a sub-population of raft-associated SERT transporters and is observed at high serotonin concentrations [9].

**Fig 6:**
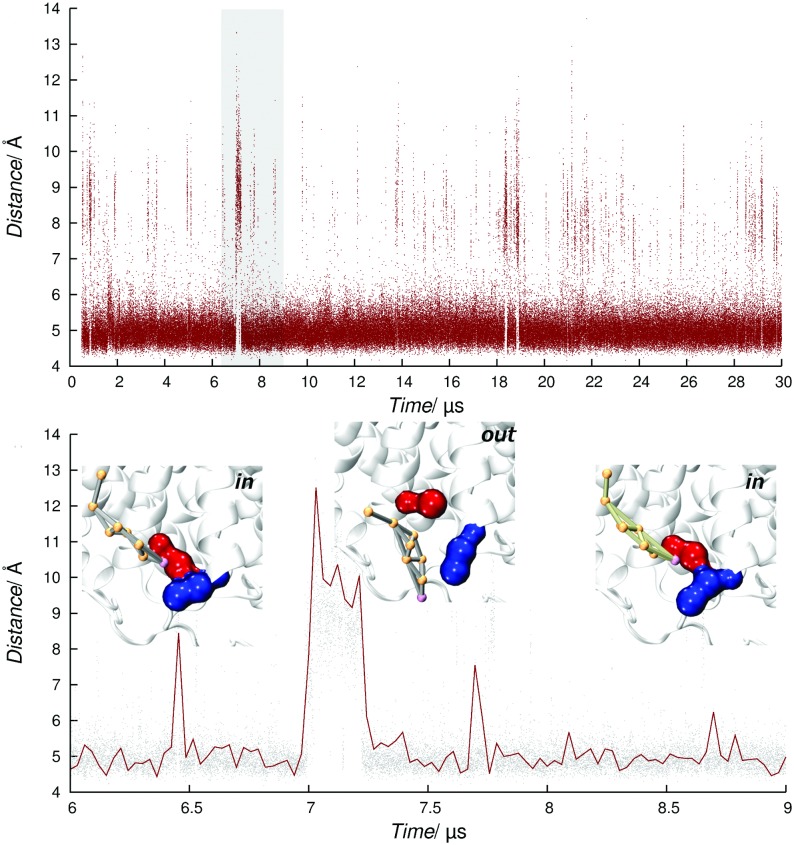
Distance between K85-D360 side chains taken from a representative trajectory of SYS3. The three insets show the two residues as blue and red surfaces in contact with cholesterol molecules (grey and green) in the time interval defined by the gray box.

A similar effect could also arise from cholesterol binding at site S, which is located in proximity of K and encompasses residues belonging to TM1a, TM7 and to the fifth intra-cellular loop (IL5) (Fig 5). Site S and K can be occupied at the same time, with distinct cholesterol molecules interacting in a tail-to-tail fashion (Fig 5). Notably, the outlier residue F531 (IL5) was associated to the highest *t*_max_. Sequence alignment shows that this residue is only conserved in NET, while in DAT it is replaced by Q514 (Fig C in S1 Supporting Information). As seen for non-linear sites, stacking between tyrosine/phenylalanine and cholesterol rings is relevant for the stabilization of the complex. This evidence led us to advance a second mechanism to modulate transporters function in a partly selective way. We investigated residues in proximity of site S, finding that cholesterol hydroxyl group was involved in stable interactions with a conserved lysine (K85, TM1a). The presence of cholesterol in this site favors the formation of a salt bridge between K85 and D360 (TM7). As reported in Fig 6 and, upon cholesterol binding, the two charged residues come in close contact (~ 5 Å), whereas much higher distances are sampled when no sterol is bound.

This behavior was observed with several distinct cholesterol molecules. Since the intracellular gating system is likely dependent on the rupture of the cytoplasmic R79 (TM1a)—E452 (IL4) salt bridge [21], we advance an additional switching mechanism whereby cholesterol could modulate SERT function by stabilizing the transporter in an outward facing state. Furthermore, mutagenesis studies have revealed the importance of outlier V366 (TM7) in preserving transport functionality [52]. Cholesterol could possibly regulate TM7 flexibility establishing long lasting interactions with V366.

**Fig 7:**
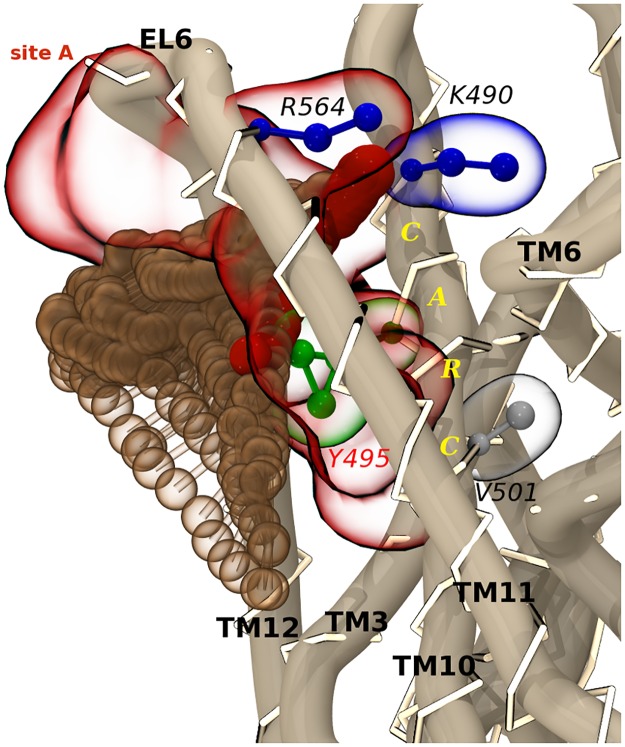
Site A in complex with a cholesterol molecule hopping between the two sub-sites A1/A2. The included CARC motif on TM10 and its key amino acids (CPK representation) follow the coloring scheme of Fig 4. Cholesterol hydroxyl group (red beads) does not interact with the K490 and V501, but H-bonds R564 on EL6.

A and C sites are still characterized by a non-linear topology, but, unlike sites K and S, they show over the course of the simulation cholesterol bound in different, but recurring, orientations (Table 3, Fig 4c, 4d, 4e, 4g and 4h). By adopting the widely accepted terminology employed in pharmaceutical chemistry, hereafter we refer to these distinct binding modalities to the same site as “multiple binding modes”, as opposed to the well-defined binding configuration shown by cholesterol in sites K and S. More in details, site A is located in a region lining the sixth extra-cellular loop (EL6). Here, cholesterol contacts helices TM10-TM12 and some of the membrane facing residues belonging to EL6 by adopting two distinct binding modes. The corresponding sub-sites, hereafter referred to as A1 and A2, share the outlier Y495 and, similarly to sites K and S, tyrosine side chain appear to play a pivotal role in the interaction with cholesterol. Direct sub-site-hopping events were observed (Fig 7).

Sub-site A1 mostly lies on TM10, (Fig 4g) where cholesterol interacts with Y495, which, together with residues on domains TM10-12, has been proposed to be involved in the allosteric modulation of the substrate binding affinity [53, 54]. Moreover, residues on TM11 contribute to van der Waals interactions through L553, L557 and P561, while R564 on EL6 establishes H-bonds with the cholesterol hydroxyl group. The primary sequence on TM10 matches a well-known cholesterol consensus sequence, the inverted CRAC motif (also called CARC), which differs from the original one by the reading direction of residues [34]. CRAC/CARC motifs have been suggested to play a role in the modulation of membrane proteins activity [55]. They are both found on transmembrane regions and are characterized by a specific series of amino acids, consisting in an apolar (V/L), an aromatic (Y/F), and a basic (R/K) residue for CRAC, spaced by one to five membrane facing residues. In the case of CARC, keeping the N- to C-term reading direction, the residues are listed in the reverse order. Recent efforts have been undertaken in the identification of non-steroidal CRAC ligands for a mitochondrial membrane protein [56], suggesting such motifs as promising targets in drug discovery. Sub-site A1 includes the motif K490-Y495-V501 (Fig 7), which is located in the upper half of TM10, and is consistent with this definition. Despite the high selection pressure on these sequences, among the homologous NSS, the mandatory basic residue in the first position of the CARC sequence (K490) is only conserved in SERT (Fig C in S1 Supporting Information), and not available for cholesterol interaction. Moreover, it is replaced with D401 in the orthologue LeuT. The crystalized inward facing conformation of LeuT highlighted that D401 makes H-bonds with the A319 backbone on EL4, contributing to loop closure during transport [21]. Even if D401 is not conserved in SERT, K490 preserves the backbone interaction with the corresponding G402 on EL4. Another functionally relevant residue found within the CARC motif is the conserved gatekeeper residue E493 (together with R104). It is tempting to speculate that specific cholesterol binding at this site could influence the residue’s availability to form a salt-bridge with R104 and thus to close the SERT extracellular gate. Interestingly, the upper half of TM10, TM1 and TM3, plus residues on EL4 and EL2, have been shown to form an allosteric pocket for (S)-Citalopram in the extracellular vestibule of SERT [57]. This secondary binding site, called S2 site, is analogous to the S2 site in LeuT [58], and has been found to bind inhibitors and a second substrate [58–61]. In fact, mutations of residues like K490, among the others on TM10, reduce SERT’s affinity for selective ligands [59, 60]. Furthermore, S2 site has been proposed as a symport effector in NSS [62, 63]. Together, these evidences led to addressing this pocket in the attempt to identify novel allosteric SERT inhibitors [64]. Similar implications were found for sub-site A2, which is associated to a cholesterol binding mode rotated of approximately 90° around Y495 towards EL6 and TM12 (Fig 4h). Besides the central tyrosine, contacting residues belonging to TM11, TM12, and EL6 are R564, Y572, I575, I576, Y579 and C580. Some of these residues line a recently identified allosteric pocket which involves, again, the extracellular portions of TM1, TM6, TM10, TM11 and EL6 [65]. Accordingly, a novel SERT allosteric modulator able to stabilize SERT in an outward facing conformation was designed. This pocket shares with sub-site A1 the CARC-belonging K490, and with sub-site A2 residues R564 and Y568, the latter being not always available for cholesterol binding. The results of our simulations are in line with the reported studies converging on TM10-TM12 and EL6 as important regions for allosteric modulation. The proximity of site A to the S2 site in LeuT, and to the novel allosteric pocket, provides an interesting clue to support the hypothesis that cholesterol can favor the outward facing state of SERT, interacting with site A from the lipid milieu.

**Fig 8:**
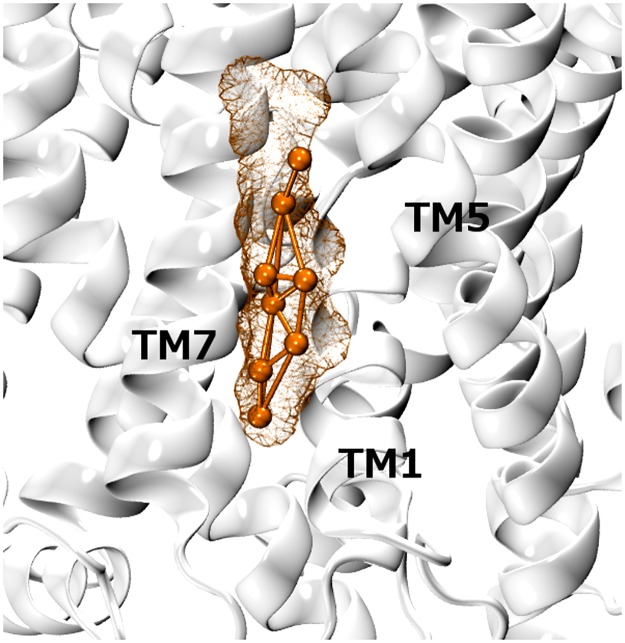
X-ray structure of *d*DAT (pdb ID: 4M48). Coordinates for the co-crystalized lipid are reported as orange wired surface and overlap with the aligned pose of a representative cholesterol molecule (orange CPK representation) emerged from simulations (the aligned coarse-grained *h*SERT structure in not shown for clarity).

The second characterized non-linear site with multiple binding modes was site C (Fig 4c, 4d and 4e). Cholesterol fits in a cleft between TM1, TM5 and TM7 and was observed to experience a highly dynamic behavior, achieving a total of three orientations. All these binding modes involve the conserved residue Y289, which is also an overlapping outlier, as well as V86, L89, L90, I93, V367, A285,S286 and T371. Fig 8 shows that one of the sampled modes nicely fits the molecular surface of a cholesterol molecule co-crystalized with the eukaryotic *d*DAT [11].

Considering the overall conservation of the crystalized cholesterol site in *d*DAT and *h*SERT, higher cholesterol densities and defined binding modes could be expected. However, the shape of SDF in site C suggested a different picture. The evidence that cholesterol experiences multiple binding modes within the same site, might represent an intrinsic functional feature of site C, but it also suggests that subtle structural differences in the site could be important to trigger a stable interaction with the sterol. In particular, a consistent pattern of substitutions of leucine/isoleucine residues on TM7 in *d*DAT with valines in *h*SERT is found (Fig 4e and Fig C in S1 Supporting Information). Mutations are conservative in terms of polarity but not in terms of volume. The presence of less bulky amino acids in SERT might lead to less defined spots in site C, compared to DAT. A site of a larger size could be responsible for inefficient van der Waals interactions and higher lipid mobility. Furthermore, S293 and T286 in SERT, which are conserved in the human DAT homologue, disrupt the hydrophobicity of site C which lodges the isooctyl side chain of cholesterol in the crystal structure (L277 and L270 in dDAT, respectively). These observations provide a reasonable explanation for the multiple binding modes and weaker SDF displayed by cholesterol at this site.

**Fig 9:**
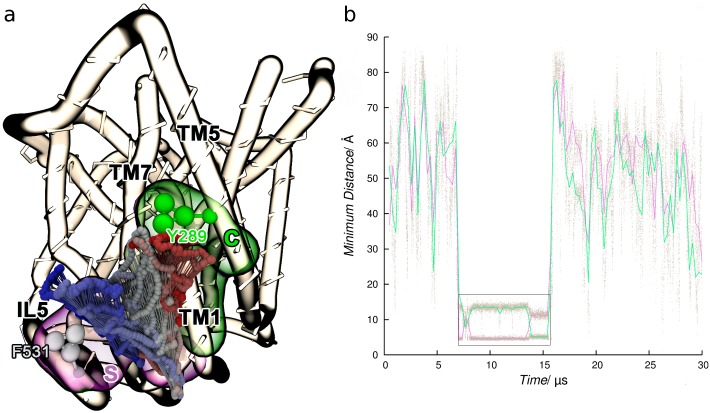
Cholesterol hopping from site S to C. (**a**) The cholesterol molecule is colored according to simulation time. (**b**) Time series of the minimum distance between cholesterol and the outliers F531 (site S, purple curve) and Y289 (site C, green curves).

In line with the latest paradigm for GPCR-cholesterol interactions [47], we also found that cholesterol adopts an intrinsically dynamic behavior at SERT surface, characterized by partial unbinding events and occasional site-hopping. In Fig 9, an example of a cholesterol molecule swapping from site S to site C is shown (see below).

Even though the presence of cholesterol seems to favor outward-open conformations, experiments have confirmed that depletion of this lipid negatively affects transport rates and affinities for substrate and Citalopram [8]. Unfortunately, MARTINI force field does not allow the investigation of major conformational changes on proteins, actually precluding the direct study of the mechanism through which cholesterol modulates serotonin transport. While atomistic simulations are required to fully characterize the role played by cholesterol on SERT transport, at the current level of theory we can speculate that cholesterol could respond to substrate release and assist conformational changes through a dynamic communication between sites K and S (tail-to-tail), and S and C (site hopping). In this respect, it is known that during the transport cycle helix TM1a undergoes an hinge like motion exposing basic residues (K85/K86) to the apolar lipid acyl chains [21]. Since the contacts between K85 and cholesterol are maintained during site hopping, it is likely that this interaction might be preserved along the transport cycle as well as in the inward-open conformation of SERT, so as to shield the charged residue from an unfavorable environment.

### Selectivity of lipids interaction sites

**Fig 10:**
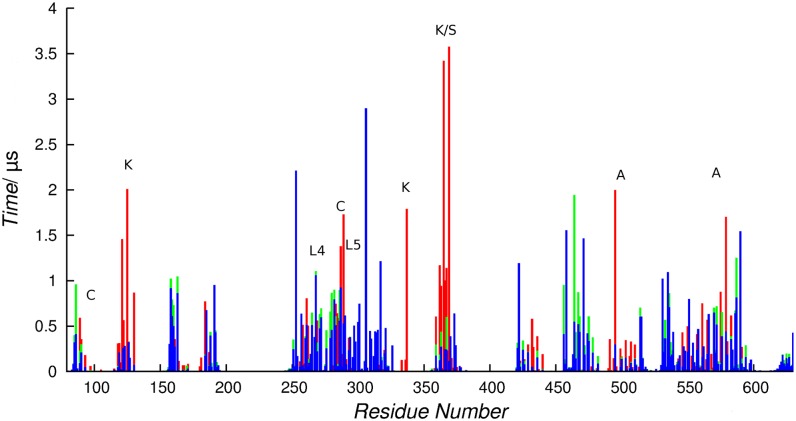
Maximum occupancy times for all lipids. DSPC (blue), POPE (green) and cholesterol (red); values were obtained averaging values collected in SYS3.1–2. Sites regions along the sequence are indicated by the corresponding names.

The selectivity of cholesterol hot-spots was derived from a combined analysis of maximum occupancy times (Fig 10) and SDF of the two phospholipid species present in our system. In agreement with previous findings [45, 66], microsecond long *t*_max_ were observed for PC and PE in SERT regions not corresponding to the identified sites. Instead, in proximity of cholesterol hot-spots, phospholipids can occasionally reach *t*_max_ of hundreds of nanoseconds, while the sterol typically shows interactions in the microsecond time scale.

Interestingly, at these sites, SDF showed negligible densities for phospholipids, indicating transient contacts established by PC and PE species during partial cholesterol unbinding events. This behavior is in line with a previously suggested cooperative effect whereby lipids might act as a sticky solvent [66], able to shape and tune the SERT-sterol interactions without actually competing with cholesterol binding. We also note that, since no PC or PE density was observed in sites K, S, C, and A, these can be defined as “non-annular” sites, identifying regions poorly or totally inaccessible from lipids other than cholesterol [67]. On the other hand, in spite of bearing a specific CRAC motif, site L4 showed the lowest selectivity for the sterol.

Another lipid modulator found in the inner leaflet of bulk and raft membranes is phosphatidylinositol 4,5-bisphosphate (PIP2) [68]. Interestingly, combined experimental and computational reports have recently suggested PIP2 to play a functional role in SERT and DAT biology [30, 31]. In particular, MD simulations performed on DAT showed that this anionic lipid can mediate the interaction between the cytosolic N-terminal domain of the transporter and the intracellular loop 4 (IL4) via electrostatic forces, attracting the former toward the inner leaflet. This mechanism would enable the kinase-dependent phosphorylation required for the amphetamine-induced reverse transport of the substrate, which is, in turn, responsible for the psychostimulant effect. Moreover, subsequent simulations on DAT have shown that PIP2 is able to trigger the inward-open state and hence ions/substrate release, by contacting, among the others, residue R443 on IL4, which is conserved in SERT (K460), and K352 (IL3) [32]. However, experimental evidence has also demonstrated that depletion of this lipid does not affect physiological substrate reuptake, neither in SERT nor in DAT [30, 31], suggesting that direct and reverse transport may be independently managed by membrane machinery [32]. Since the effect of this lipid on substrate reuptake is still debated, PIP2 was not considered in our membrane model environments. It is nonetheless informative to check the maximum occupancy times for cholesterol and phospholipids in SERT residues that are known to be implicated in PIP2 contacts. From the four data-sets belonging to SYS3, we obtained *t*_max_ values very close or even lower to the median, indicating that none of the lipids in our system significantly contacted those basic residues. More in details, K352 is not contacted by cholesterol in SYS3, whereas for the population reported in Fig 2a, a*t*_max_ value of 1.6 ns was obtained. A slightly higher average value was observed for K460, (*t*_max_ = 6.9 ns), while in the merged population a value of 13.8 ns was recorded. Similarly, POPE interacted with K352 and K460 with average values of 1.9, 45 ns, while DSPC returned values of 5 and 38 ns, respectively, indicating that these lipids are unlikely to compete with PIP2 binding sites.

## Conclusions

Concluding, we developed an efficient protocol of general applicability to be used in the study of lipid-protein interactions. This coarse-grained-based approach can return relevant insights in a reasonable amount of calculation time. In turn, these insights can prompt further and more detailed analyses at a fully atomistic level of detail. In this case, the reported protocol was applied to SERT. Our simulations revealed six potential interaction sites between cholesterol and the transporter. While an increasing number of studies focuses on cholesterol modulatory role in channels and GPCRs, to the best of our knowledge, this is the first computational report on the interaction between cholesterol and a member of the NSS family. Within the limits of a study carried out on homology models, which even when a strongly homologous template is available can be less than ideal for recapitulating all interaction details, [69] several intriguing elements emerged. First, all identified sites were located in areas strongly implicated in the regulation of transport activity. Here, we proposed a mechanism through which cholesterol binding could promote chloride coordination and protein activation, and we also postulated a role in the gating mechanism for the K85-D360 pair, suggesting that this interaction can be affected by cholesterol binding. Second, our simulations successfully reproduced the binding mode of a cholesterol molecule as found in a recently released DAT crystal structure. Finally, we characterized the topology of two not yet reported functional CRAC and CARC sequences, and we speculated of the involvement of the latter in allosteric modulation and the former in the poorly understood oligomerization process (see S1 Supporting Information). In line with the spirit of the proposed approach, this comprehensive description of hot-spots on the SERT surface generated at the coarse-grained level paves the way to more quantitative atomistic studies and hopefully, in due time, to the discovery of allosteric modulators of the transporter.

### Note

While this manuscript was under submission, the first crystal structure of human SERT has been published by Coleman and co-workers [70]. Even though non-negligible structural differences were found between hSERT and bacterial LeuT, the overall fold is generously preserved in the two proteins, especially in the first eight transmembrane helices. Among the most remarkable discrepancies, we highlight those observed in the terminal region (TM9-12), which also showed unexpected differences when compared to the analogous portion of the eukaryotic *d*DAT. Another unexpected feature of hSERT relates to the dimeric assembly found in crystal lattice, which has been defined an “apparent dimer”, since membrane-spanning regions of single monomers do not match each other. Conversely, there is clear experimental evidence that TM12 partakes in oligomer formation [71]. A Cholesterol molecule was found to interact at a linear site on the extracellular portion of TM12, i.e. TM12a, with W573, I576, L577 and a surface exposed cysteine residue (C580), which was mutated in alanine in SERT construct. Simulating the wild-type transporter, sterol occupancy in such region is not expected to match the crystallographic one [72]. However, we found that cholesterol is able to contact C580 and I576 when bound to the interaction site A (sub-site A2) identified in this study. This region contains a known cholesterol binding motif (CARC) and lines the allosteric Citalopram binding site in the *h*SERT crystal structure. Such findings demonstrated that our protocol could have indeed successfully identified hot-regions for cholesterol binding nearby helices involved in oligomerization and allosteric regulation of the transporter.

## Supporting Information

S1 Supporting Information Text file including additional data and all supporting Figures and Tables. The file contains also a discussion on the use of the *h*SERT homology model, an expanded section on statistical methods and the characterization of linear sites L4 and L5.
(PDF)
