# Supplementary material for: Mapping Cholesterol Interaction Sites on Serotonin Transporter through Coarse-Grained Molecular Dynamics
Source: PLoS One. 2016 Dec 1;11(12):e0166196. doi: 10.1371/journal.pone.0166196 (PMC5132266; doi:10.1371/journal.pone.0166196)
Supplement: S1 Supporting Information — The file contains also a discussion on the use of the hSERT homology model, an expanded section on statistical methods and the characterization of linear sites L4 and L5. (PDF) [file pone.0166196.s001.pdf]

Supporting Information

Mapping Cholesterol Interaction Sites on Serotonin Transporter through Coarse-Grained Molecular Dynamics Simulations.

Mariarosaria Ferraro, Matteo Masetti,* Andrea Cavalli, Maurizio Recanatini,

Giovanni Bottegoni*

Table of content

1. ON THE USE OF LeuT AS A TEMPLATE TO MODEL *h*SERT. S3

2. EXPANDED METHODS S5

2.1 COARSE-GRAINED MODELLING, SELF-ASSEMBLY AND SIMULATIONS SETUP S5

2.2 STATISTICAL ANALYSIS OF MAXIMUM OCCUPANCY TIMES (RESIDUE-BASED ANALYSIS) S6

2.3 ASSESSING SAMPLING CONVERGENCE S8

3. TABLES A AND B S10

4. FIGURES A-D S12

5. CHARACTERIZATION OF LINEAR SITES L5 AND L4 S16

5.1 EFFECT OF CHOLESTEROL CONCENTRATION ON LINEAR SITE OCCUPANCY S17

6. REFERENCES S18

1. ON THE USE OF LeuT AS A TEMPLATE TO MODEL *h*SERT.

Here we discuss on the implications of using LeuT as a template for *h*SERT related to the structure similarity of the two transporters, and to their oligomerization state.

To quantify the impact of the LeuT-based model of *h*SERT in the regions of inference, we calculated the Root Mean Squared Deviation (RMSD) from two among the latest published *d*DAT structures in complex with dopamine (PDB id: 4XP1) and with a substrate analog (PDB id: 4XPA) [1]. It has to be noticed that *d*DAT bound to its substrate displays an outward-open conformation [1], which overlaps with the state achieved by the transporter in complex with nortriptyline (PDB id: 4M48) [2]. On the contrary, 4XPA crystal complex shows a partially occluded state closer to the LeuT template (PDB id: 2A65) used to model SERT [3].

The structural comparison was limited to the TM regions of the transporters (fragments: 86-111; 116-143; 159-189; 254-271; 275-301; 324-350; 358-386; 420-453; 463-480; 488-515; 536-559; 575-599 in *h*SERT). The corresponding residues in the two *d*DAT structures were used to align different groups of helices and obtain a comparative set of RMSDs. Unsurprisingly, superimposing the whole SERT TM portions with 4XP1 and 4XPA crystal structures, RMSDs of 3.1 Å and 3 Å were obtained, respectively. By excluding the kinked helix TM12, both crystals deviate from SERT homology model of 1.9 Å and 1.8 Å, respectively, so that the contribution of this helix to the average deviation is approximately of 1 Å. To further estimate the weight of the peripheral TM12 in the overall RMSD calculation, we repeated the analysis excluding TM11, getting unvaried values. Based on these results, we focused on 4XPA for further calculations. From a in depth inspection of the occluded states in LeuT and *d*DAT, the extracellular portions of TM1 and TM6, i.e. TM1b and TM6a, appear to be more outward-facing in *d*DAT relatively to LeuT [1]. As such portions are sequestered in the core and are not exposed to cholesterol, we assessed the RMSD excluding TM1b (residues 99-111) and TM6a (residues 324-337), besides TM12. This choice is additionally supported by the fact that the helicity of TM1 and TM6 is broken in the mid-section, allowing independent hinge-like motions. The RMSD calculated aligning TM1a, TM2-5 and TM6b-11 to 4XPA structure returned a minimum value of 1.7 Å (Fig A), which support the use of the provided SERT model in our simulations, and reveal a noteworthy overall conservation of functional domains between orthologous transporters. Although the biological role of oligomerization is still subject of intense studies [4, 5], formation of dimers

and higher-order assemblies has been widely recognized as a featuring property of SERT [6], DAT [7] and many other NSS family members [4]. In this respect, fluorescence-based and mutational studies have proposed TM1, TM2, TM6, TM11 and TM12 as potential oligomeric interfaces with a role in SERT expression or activity [8-10]. Despite interactions between protomers affect transport [11], it has been shown that monomer is fully active and coexists on cells' surface with stably expressed higher-order oligomers [12-14].

Until very recently, none of the eukaryotic crystal structures contained information about biologically relevant NSS assemblies [1, 2, 15], being the bacterial LeuT the only NSS member displaying a dimeric arrangement in crystal lattice [16]. Hence, in order to study *h*SERT dynamics within a plausible oligomeric environment, dimers modeled from LeuT have been generated and widely simulated in MD, showing well agreement with experimental data [17-19]. No influences on SERT function due to the chosen arrangement have been reported in these works, except for a study on LeuT [20], where a depression in the lipid bilayer observed in monomeric system justified the use of the dimeric structure.

The choice of LeuT as a template to model SERT dimeric assembly has been globally supported by experiments showing that TM12, which forms an interface in LeuT is likewise involved in *h*SERT oligomeric contacts [10]. Notably, the N-terminal portion of TM12 (TM12a) contains a known GxxxG (G578-x-C580-x-G582) dimerization motif found in Glycophorine A [21]. According to the covalent nature of SERT oligomers [22], the surface exposed C580 (enclosed in the motif) could potentially play a role in formation of disulfide bonds.

Unlike LeuT, eukaryotic DAT from *Drosophila M.* is monomeric in crystals and present a featuring kink in the terminal portion of TM12 (i.e. TM12b), which originates from a proline residue not conserved in LeuT [1, 2, 15]. This structural difference has opened a debate on the role of TM12 in NSS oligomer formation among the eukaryotes, leading to contrasting conclusions. While Penmatsa et Al. have disfavored the kinked TM12 to be a reasonable contacting surface in eukaryotic transporters [2], Stockner et Al. have proposed that this kink could be stabilized thanks to the involvement of TM12 in oligomer formation [23]. Further studies are crucial to solve this controversy; MD simulations could establish a link between static crystal structures and experiments, which indeed identify TM12 as an oligomerization domain in hSERT [10]. Given such findings, the involvement of TM12 in the interface of the "apparent dimer" (see the Main text)

observed in the very recent *h*SERT crystal structure should deserve attention and stimulate further investigation [24].

2. EXPANDED METHODS

2.1 COARSE-GRAINED MODELLING, SELF-ASSEMBLY AND SIMULATIONS SETUP

In the latest MARTINI parameterization [25], atoms of each residue are typically mapped into two to five beads. There are four main types of particles: polar (P), nonpolar (N), apolar (C), and charged (Q). Each particle is further characterized according to hydrogen-bonding capabilities and its degree of polarity. The smooth potential resulting from the grouping strategy allows to observe events such as the self-assembly of protein-membrane systems in a computationally efficient way. In this approach, random configurations of protein, lipids, and water can be effectively evolved toward defect free bilayers.

The SERT monomer was replicated using ICM software to obtain a dimeric LeuT-like structure [26]. The atomistic coordinates for SERT were changed in coarse-grain ones through the script *martinize.py* available from the MARTINI web site (http://md.chem.rug.nl/). To retain secondary and tertiary structure, an elastic network was used within a cutoff of 9 Å and with a force constant of 500 $kJmol^{-1}nm^{-2}$. Even though the elastic network prevents large conformational changes, here we were mostly interested in characterizing cholesterol hot-spots at the protein surface in a given SERT conformation, which was representative of a specific state along the transport cycle.

Lipid ternary mixtures used in SYS1, SYS2 and SYS3 contained 1,2-di-stearoyl-sn-3-glycero-phosphocoline (DSPC), 1-palmitoyl-2-oleoyl-sn-3-glycero-phosphoethanolamine (POPE) and cholesterol molecules in the molar ratios of 50:35:15, 50:30:20, and 50:25:25, respectively, for a total of 512 lipids per system (we refer to our previous work for details on lipids CG models) [27]. Then, the SERT model was incorporated into the raft-like membrane through the self-assembly procedure developed by Bond and Sansom [28]. The protein was first placed at the center of a cubic box (13x13x13 nm) and then solvated with lipids and water corresponding to the lipids' number of hydration in a randomly reiterated procedure as implemented in the *genbox* routine. The so-obtained systems were minimized with 10,000 cycles of steepest descent. After this step, water was added to completely solvate the system. Self-assembly was carried out at

300 K under isotropic pressure control. Periodic boundary conditions were employed to minimize finite size effects; neighbor list cut-off was set to 1.4 nm, and a time step of 20 fs was used. Non-bonded interactions were treated in line with the MARTINI model standard protocol [29]. Temperature and pressure were controlled using the Berendsen weak coupling algorithm [30]. Pressure coupling and compressibility were 40 ps and 1e-05/bar, as suggested by Bond [28]. Dumping time for writing trajectories was 200 ps. The self-assembly production run were 200 ns long, during which systems spontaneously evolved into a bilayer. The assembled systems were then equilibrated for other 200 ns switching from isotropic to semi-isotropic pressure control. Structural and diffusion parameters were in line with the previously validated protein-free membrane models [27]. Finally, we evolved each copy for 30 μs of simulation at 300 K for an aggregate time of 120 μs per system. Analyses were carried out discarding the first 500 ns of each trajectory, to avoid any memory of the initial state.

2.2 STATISTICAL ANALYSIS OF MAXIMUM OCCUPANCY TIMES (RESIDUE-BASED ANALYSIS)

The maximum occupancy times were collected for 466 individual residues, including TM regions and membrane facing loops (except EL2 and EL4), within 6 Å of cholesterol, for both a and b monomers of each simulated system. D'agostino-Pearson test was performed on each of the resulting data-set to assess deviations from normality [31, 32], whereas the departure of skweness and kurtosis from a normal behavior was evaluated through the K2 omnibus test. When the distribution is normal, the value of K2 approximates a χ^2 (chi-squared) curve with two degrees of freedom. A p-value for K2 test is an indirect estimation of the probability that deviations from the expected profile are not simply due to chance. The hypothesis of normality can be accepted or rejected if p-value is greater or lower than the chosen level of significance (α), respectively. If the distributions approximate a normal curve, parametric statistics and the related T-test or Z-test can be applied to identify outliers residing out of the standard confidence intervals (95% - 99%) [33]. When dealing with non-normal curves, non-parametric methods are necessary to infer statistical hypothesis between samples [34]. In this work, in which specific interactions could reasonably return t_{max} three orders of magnitude higher than the remaining population, a non-normal behavior was expected and properly verified as described above. Non-normal distributions are usually visualized through a boxplot representation. The sample ordinal data are divided in quartiles providing an indication of data spread. Interquartile range (IQR)

is calculated as the difference between the 3rd and the 1st quartile and it is a meaningful index of spread which allows to identify the outlier region. A value that falls above the threshold given by:

$$T = 1.5 \times (\mathrm{IQR}) + 3^{\mathrm{rd}}\ \mathrm{quartile} \quad (1)$$

is usually considered an outlier [35].

Having a total of four data-sets per cholesterol concentration, we carried out statistical tests of hypothesis by comparing the medians of distributions in order to assess whether the sets could be grouped in a unique macro-sample. The advantage of merging trajectories of statistically identical copies is that increasing the amount of sampling the statistical analysis becomes more robust. Hence, t_{max} can be extracted from more representative samples and single outliers can be uniquely identified for each SYSX system. Furthermore, this approach also allowed us to identify anomalous data collected on individual data-set.

A Kruskall-Wallis test for independent non-normally distributed samples was performed [36]. This is equivalent to the ANOVA test for more than two non-normally distributed populations and establishes a comparison with the tabulated test statistics, χ^2. Instead of comparing population means, this method compares population mean ranks. The null hypothesis is that the population medians are equal, versus the alternative hypothesis that there is a difference between at least two of them. The values of t_{max} collected on the four data set of each SYSX system were combined and ranked in ascending order of magnitude. Tied values were assigned the average ranks. The Kruskall-Wallis test is based on the equation of test statistics H in presence of tied values:

$$H = \frac{1}{S^2} \sum_{j=1}^{m} \frac{R_j^2}{n_j} - \frac{N(N+1)^2}{4} \quad (2)$$

where,

$$S^2 = \frac{1}{N-1} \left\{ \sum_{j=1}^{m} \sum_{i=1}^{n_j} R_{ij}^2 - \frac{N(N+1)^2}{4} \right\} \quad (3)$$

In Equation (2) and (3), j is the number of samples, n_j is the sample size in j-th sample, N is the total samples size. The value R_j is the sum of ranks for the j-th sample. The so defined function H approximates

the χ^2 distribution. At a significance level of 0.01 and with (j – 1) degrees of freedom, the H test must adopt a value lower than the corresponding limit value for significance in the χ^2 curve. Dealing with four samples, the tabulated reference value was 11.34.

To verify the existence of a significant difference (due to cholesterol content) between the three macro samples obtained in the previous step, the same Kruskall-Wallis test was applied. Dealing with three samples, the tabulated reference value is 9.21. A value above the threshold indicates statistical evidence that cholesterol amounts has an effect on t_{max} distributions. Hence, statistically significant residues which overlapped among the three systems should be selected. This is often a good strategy to increase the robustness of the outliers, since they must be located in the tail of the three distributions irrespectively of changes in cholesterol enrichment. If the H test is lower than the reference value, the hypothesis that the tree macro systems come from a unique population cannot be rejected, implying that the applied changes in molar ratios in the simulated systems do not alter the distribution of t_{max} from a statistical point ofview. In other words, maximum occupancy times per residue collected in SYS1 are not significantly different from those observed in the most concentrated SYS3, so t_{max} can be recalculated and outliers can be extracted from a single population (properly checked for normality) obtained merging the whole statistics of the three macro samples.

2.3 ASSESSING SAMPLING CONVERGENCE

In order to verify the exhaustiveness of sampling achieved using four data-set per SYSX, we assembled a third independent copy of SYS3 (SYS3.3) reaching a total of six independent data-set. Differences in the two macro-samples (four vs. six data-set) were assessed only in SYS3. The reason for this choice resides in two competing effects related to cholesterol content of the simulated systems, which are crucial to assess the sampling convergence and discussed in the Results and Discussion section of the Main Text.

The six data-sets obtained with SYS3 were analyzed as described in section 2.2 and subjected to a first comparison of their medians via the Kruskall-Wallis test (χ^2 = 15.09, α = 0.01). This was useful to compare the data homogeneity within one or more replicas. Samples were then grouped in SYS3.1-2 and SYS3.1-2-3 to perform the Wilcoxon Rank Sum test for non-normal samples [37]. Here, we checked whether the addition of a third system was able to statistically change spread and location of the distribution obtained

from the original setup. This non-parametric test is the equivalent of the t-test for normal curves: maximum occupancy times per residue were merged and ranked in an increasing ordered distribution. Rank values belonging to the sample of smallest size ($n1$ or $n2$) were then summed together to obtain our test statistics T. Given the large size of the samples (356 and 359 for SYS3.1-2 and SYS3.1-2-3, respectively), we can assume that T is approximately normal and recall the Z-score to assess the equality of the two samples. The Z-score is given by:

$$Z = \frac{T - E(T)}{\sigma(T)} \tag{4}$$

where E(T) is the mean of ranks distribution,

$$E(T) = \frac{n1(N+1)}{2} \tag{5}$$

and σ(T) its standard deviation,

$$\sigma(T) = \frac{\sqrt{n1n2(N+1)}}{12} \tag{6}$$

with N being the total samples size. The obtained Z-score was associated to the probability under the Gaussian curve (p-value) and compared with the chosen level of significance ($\alpha = 0.01$). A p-value higher than α establishes no significant differences in the two populations, implying that, inclusion of two additional monomers does not statistically influence the spread and location of the population we already obtained with four monomers. In the opposite case, at least triplicated copies of the systems should be considered to increase confidence in results.

3. TABLES A AND B

Table A. Results of D'agostino-Pearson normality test performed on single data-sets and merged distributions as described by statistical tests of hypothesis (see Models and Methods in the main text). The omnibus K2 test and the p-values are reported. K2 values approximating 0 are usually associated to normal curves. Under the assumption that the null hypothesis of normality is true, P-values indicate the probability to be wrong if the alternative hypothesis of non-normality is accepted. At the chosen level of significance (α=0.01), p-values showed to be several orders of magnitude lower than α, so that a normal behavior cannot be inferred.

Samples	K2 omnibus test	p-value
SYS1.1-SYS1.2	399.8	10^{-87}
SYS1.1a	469.6	10^{-102}
SYS1.1b	481.7	10^{-105}
SYS1.2a	371.9	10^{-81}
SYS1.2b	413.1	10^{-70}
SYS2.1-SYS2.2	422.3	10^{-92}
SYS2.1a	249.6	10^{-55}
SYS2.1b	411.9	10^{-90}
SYS2.2a	462.4	10^{-101}
SYS2.2b	171.5	10^{-38}
SYS3.1-SYS3.2	390.5	10^{-85}
SYS3.1a	383.8	10^{-84}
SYS3.1b	416.8	10^{-91}
SYS3.2a	452.6	10^{-99}
SYS3.2b	312.6	10^{-68}
SYS3.3a	390.8	10^{-85}
SYS3.3b	325.5	10^{-71}

SYS3.1-SYS3.2-SYS3.3	386.3	10^{-84}
SYS1-2-3	407.5	10^{-89}

Table B. Values of the test statistics *H* for the possible triplets obtained merging three out of the four sample monomers belonging to SYS2. We refer to the 2 monomers in SYS2.1 as 1a and 1b, whereas monomers from the copy system SYS2.2 are identified by 2a and mono-2b

**Sample combination**	1a-1b-2a	1b-2a-2b	1a-2a-2b	1a-1b-2b
**Test statistics *H***	19.28	5.75	18.54	9.80

4. FIGURES A-D

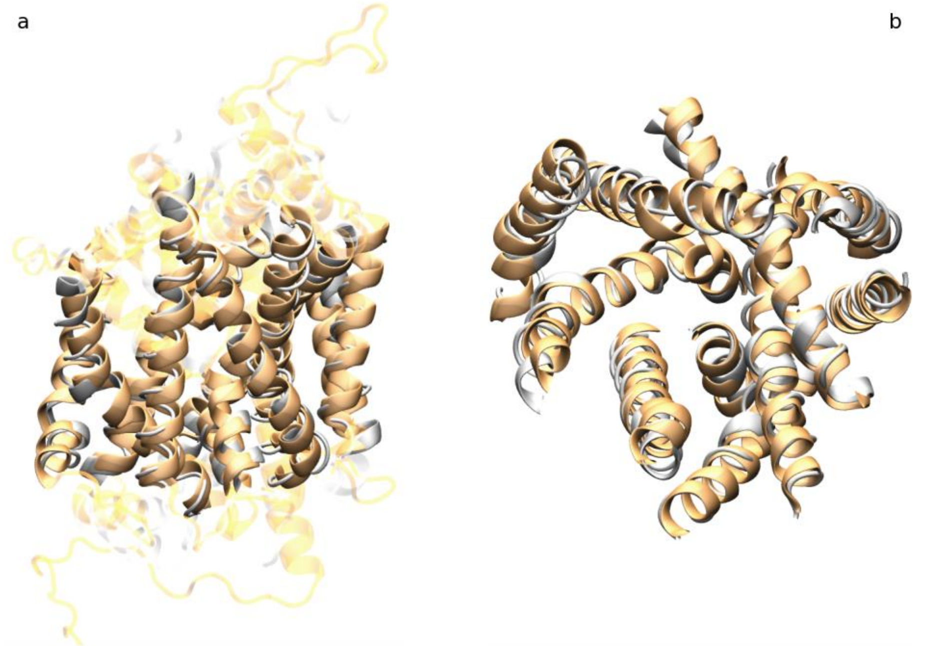

Fig. A. Comparison between protein structures. Celik's SERT homology model (orange) aligned to the TM portions of *d*DAT monomer in a partially-occluded state (silver) (PDB id: 4XPA). Panel **a**: side view of the whole monomers aligned through helices TM1a, TM2-5 and TM6b-11; extra- and intracellular portions are colored accordingly (transparent material). Panel **b**: top-view focus on the TM region only. The RMSD calculated for the aligned portions is 1.7 Å.

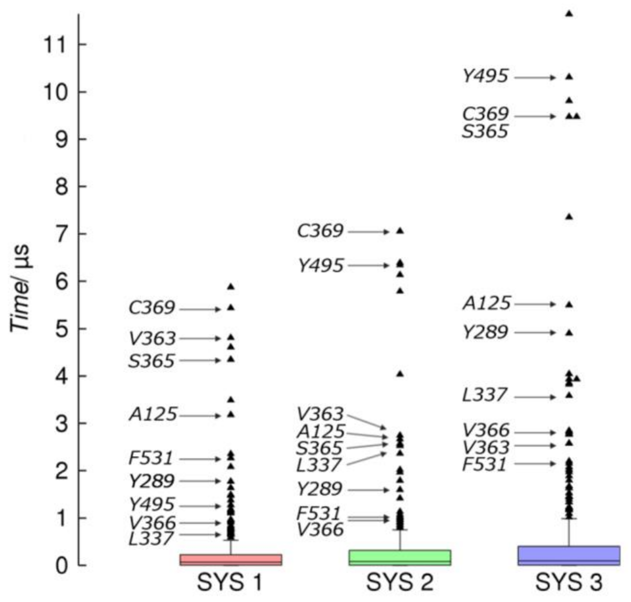

Fig. B. Maximum occupancy time per residue. Values are reported as boxplot diagrams for macro-samples SYS1-3. Robust outliers reproduced among the the three macro systems are also shown.

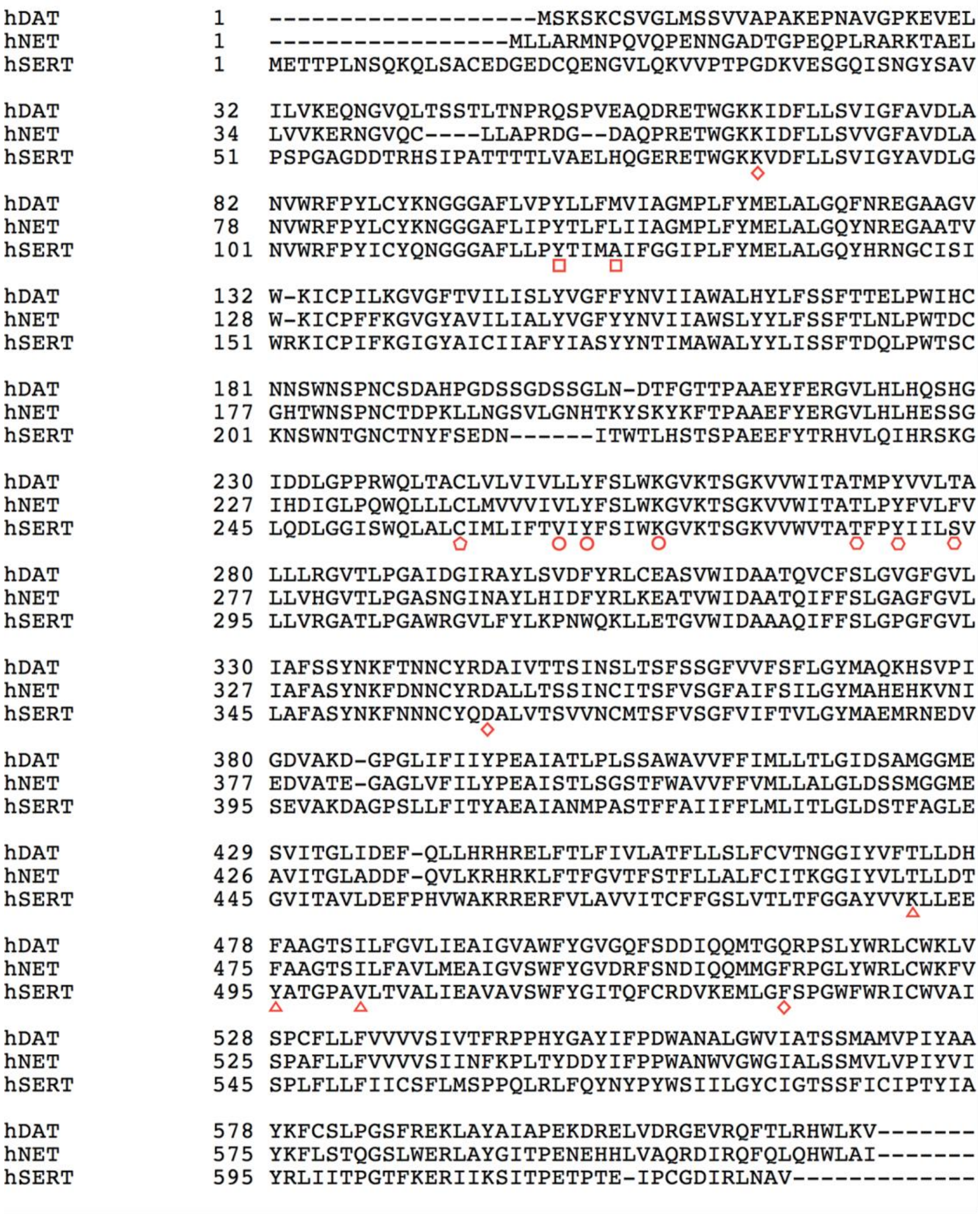

Fig. C. Sequence alignments. Amino acid sequence alignment of human serotonin transporter (hSERT) with the related neurotransmitter transporters for norepinephrine (hNET) and dopamine (hDAT), obtained using the software ICM.[26] Residues discussed in the text are marked according to the interaction site they belong to. Square, site K. Rhombus, site S. Triangle, CARC motif in site A. Hexagon, site C. Circles, CRAC motif on site L4. Pentagon, conserved cysteine on TM4 involved in oligomerization.

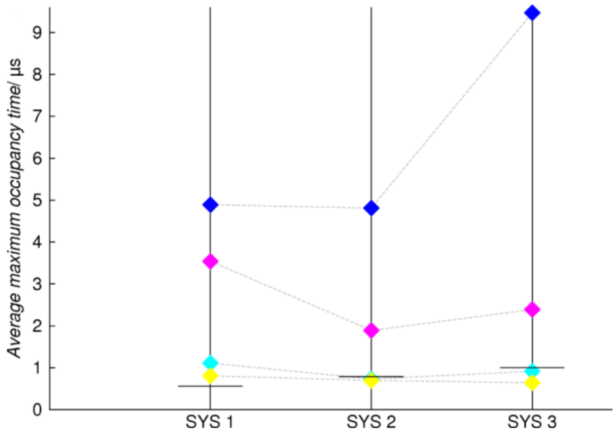

Fig. D. Maximum occupancy times at linear sites. Comparison between maximum occupancy times averaged on two representative outliers for non-linear sites, K (blue) and S (pink), and linear sites, L4 (cyan) and L5 (yellow), within the 3 systems. Time thresholds are represented with black segments and summarized in Table 2 in the Main text. Linear and non-linear sites showed very different profiles depending on their topological features which appeared to be important for determining site local responsiveness to increasing cholesterol amounts.

5. CHARACTERIZATION OF LINEAR SITES L5 AND L4

Concerning sites showing linear topologies, site L5, located on TM5 (I290-K298), displays the most interesting features. Indeed, TM5 was found to adopt an unusual helix orientation in membranes, which is reminiscent of the so-called tilted-peptides. Tilted-peptides are fragments characterized by bilayer insertion angles deviating by more than 30° from the membrane normal, and showing an increasing hydrophobicity along the axis, which makes a parallel orientation very unlikely [38, 39]. These helix segments have shown to interact with cholesterol as in the case of α-sinuclein [40]. Showing a decreasing hydrophobicity from N- to C-term ending of the sequence, and a tilt angle close to 35°, TM5 does not strictly fulfill requirements of a tilted peptide [39]. Still, highly consistent cholesterol densities were obtained in site L5. Moreover, cholesterol was found to interact with this segment in an orientation parallel to the bilayer normal and this is in agreement with what expected for tilted peptides [40], where the sterol binding mitigates unfavorable contacts established with other phospholipids. Since TM5 undergoes a large scale conformational rearrangement to reach the inward-open state (tilting of about 17°) [41], it is likely that cholesterol binding could act as a suitable interface between this helix and surrounding phospholipids, thus reducing membrane distortions that would otherwise prevent the conformational transition.

Finally, we analyze site L4, which is located on TM4 and involves residues such as V265, Y267, and K272. These residues are well conserved among SERT, DAT and NET (see Fig C) and are consistent with a CRAC sequence. As observed in the 3 somatostatin receptor, binding at CRAC sequences could be very dynamic, allowing for different cholesterol orientations around the conserved motif in order to optimally fit to the protein structure [42]. Indeed, we observed cholesterol interacting with the neighboring F268 rather than Y265, which, however, remains mandatory in the definition of the CRAC motif. We found less selectivity for cholesterol at site L4 (see Fig 10 in the Main text). However, a possible role of L4 in the formation of higher order oligomers cannot be excluded. Interestingly, TM4 has been associated to an additional interface for oligomerization in DAT. The possibility that DAT exists as dimers of dimers was investigated and confirmed by cysteine cross-linking studies involving C243 on TM4 [7]. This residue was identified as part of a secondary oligomerization interface, which allows the formation of tetramers via a disulfide bond. Similarly, SERT has been observed to coexist in higher order oligomers in plasma membrane [14], but related interfaces in SERT are still poorly understood. Here, we highlight the presence of a

conserved cysteine in this position, corresponding to C258 in SERT and in C240 in NET (see Fig C). At the same time, TM4 contains site L4, which is located in the lower leaflet of the membrane, in proximity of this conserved residue. Cholesterol is known to play a role in the oligomerization process [43, 44]. The analogies with homologues transporters and the presence of a CRAC motif, provide an interesting detail to further investigate the possibility that site L4 could be part of the oligomerization interface in SERT. This hypothesis is consistent with the poor selectivity and specificity observed for this site in our simulations. Indeed, if this interface was involved in an oligomerization process, the site would become unreachable by lipids other than cholesterol. This would represent an example where changes in the oligomeric state of a protein could modulate specificity and selectivity for a potential cholesterol interaction site.

5.1 EFFECT OF CHOLESTEROL CONCENTRATION ON LINEAR SITES OCCUPANCY

From our simulations, two of the six cholesterol interaction sites, the linear sites L4 and L5, showed consistent densities but no overlapping outliers among the three systems. An in depth analysis of maximum occupancy time (t_{max}) extracted from the three macro-samples (see Table 2 in the Main Text) provided us a rationale for this interesting behavior. We tracked fluctuations of the average maximum occupancy times for two residues in the corresponding sites L4 (V265/F268) and L5 (S293/V294), among the three systems. Next, we compared them with t_{max} for a couple of robust outliers belonging to the best ranked sites S (V363/F531) and K (S365/C369). We noticed that, even though a couple of outliers for each linear site appeared in SYS1, only one was kept in SYS2, while none of them exceeded the threshold in SYS3 (see Fig D). In contrast, robust outliers in sites S and K were located well above the threshold irrespectively of the sterol enrichment. The observed trend and time-scales of the average t_{max} recorded for sites L4 and L5 allowed us to exclude that their consistent SDFs was due to chance or multiple short-lived interactions. On the contrary, this overall decrease in t_{max} could be ascribed to the shape of the site and to cholesterol competition effect [45]. Indeed, linear sites on single helices are more susceptible to local membrane environment compared to non-linear ones because of their increased accessibility to lipids. This feature may potentially increase cholesterol competition for binding, reducing t_{max} from SYS1 to SYS3, but still preserving microsecond time-scales as well as reproducible SDFs.

6. REFERENCES

1. Wang, K. H., Penmatsa, A. and Gouaux, E. 2015. Neurotransmitter and psychostimulant recognition by the dopamine transporter. *Nature*. **521** (7552): 322-327.

2. Penmatsa, A., Wang, K. H. and Gouaux, E. 2013. X-ray structure of dopamine transporter elucidates antidepressant mechanism. *Nature*. **503** (7474): 85-90.

3. Celik, L., Sinning, S., Severinsen, K., Hansen, C. G., Moller, M. S., Bols, M., Wiborg, O. and Schiott, B. 2008. Binding of serotonin to the human serotonin transporter. Molecular modeling and experimental validation. *J Am Chem Soc*. **130** (12): 3853-3865.

4. Sitte, H. H., Farhan, H. and Javitch, J. A. 2004. Sodium-dependent neurotransmitter transporters: oligomerization as a determinant of transporter function and trafficking. *Mol Interv*. **4** (1): 38-47.

5. Sitte, H. H. and Freissmuth, M. 2003. Oligomer formation by Na+-Cl--coupled neurotransmitter transporters. *Eur J Pharmacol*. **479** (1-3): 229-236.

6. Schmid, J. A., Scholze, P., Kudlacek, O., Freissmuth, M., Singer, E. A. and Sitte, H. H. 2001. Oligomerization of the human serotonin transporter and of the rat GABA transporter 1 visualized by fluorescence resonance energy transfer microscopy in living cells. *J Biol Chem*. **276** (6): 3805-3810.

7. Hastrup, H., Sen, N. and Javitch, J. A. 2003. The human dopamine transporter forms a tetramer in the plasma membrane: cross-linking of a cysteine in the fourth transmembrane segment is sensitive to cocaine analogs. *J Biol Chem*. **278** (46): 45045-45048.

8. Sato, Y., Zhang, Y. W., Androutsellis-Theotokis, A. and Rudnick, G. 2004. Analysis of transmembrane domain 2 of rat serotonin transporter by cysteine scanning mutagenesis. *J Biol Chem*. **279** (22): 22926-22933.

9. Horschitz, S., Lau, T. and Schloss, P. 2008. Glycine residues G338 and G342 are important determinants for serotonin transporter dimerisation and cell surface expression. *Neurochem Int*. **52** (4-5): 770-775.

10. Just, H., Sitte, H. H., Schmid, J. A., Freissmuth, M. and Kudlacek, O. 2004. Identification of an additional interaction domain in transmembrane domains 11 and 12 that supports oligomer formation in the human serotonin transporter. *J Biol Chem*. **279** (8): 6650-6657.

11. Kilic, F. and Rudnick, G. 2000. Oligomerization of serotonin transporter and its functional consequences. *Proc Natl Acad Sci U S A*. **97** (7): 3106-3111.

12. Kocabas, A. M., Rudnick, G. and Kilic, F. 2003. Functional consequences of homo- but not hetero-oligomerization between transporters for the biogenic amine neurotransmitters. *J Neurochem*. **85** (6): 1513-1520.

13. Horschitz, S., Hummerich, R. and Schloss, P. 2003. Functional coupling of serotonin and noradrenaline transporters. *J Neurochem*. **86** (4): 958-965.

14. Anderluh, A., Klotzsch, E., Reismann, A. W., Brameshuber, M., Kudlacek, O., Newman, A. H., Sitte, H. H. and Schutz, G. J. 2014. Single molecule analysis reveals coexistence of stable serotonin transporter monomers and oligomers in the live cell plasma membrane. *J Biol Chem*. **289** (7): 4387-4394.

15. Penmatsa, A., Wang, K. H. and Gouaux, E. 2015. X-ray structures of Drosophila dopamine transporter in complex with nisoxetine and reboxetine. *Nat Struct Mol Biol*. **22** (6): 506-508.

16. Yamashita, A., Singh, S. K., Kawate, T., Jin, Y. and Gouaux, E. 2005. Crystal structure of a bacterial homologue of Na+/Cl--dependent neurotransmitter transporters. *Nature*. **437** (7056): 215-223.

17. Sinning, S., Musgaard, M., Jensen, M., Severinsen, K., Celik, L., Koldso, H., Meyer, T., Bols, M., Jensen, H. H., Schiott, B. and Wiborg, O. 2010. Binding and orientation of tricyclic antidepressants within the central substrate site of the human serotonin transporter. *J Biol Chem*. **285** (11): 8363-8374.

18. Koldso, H., Noer, P., Grouleff, J., Autzen, H. E., Sinning, S. and Schiott, B. 2011. Unbiased simulations reveal the inward-facing conformation of the human serotonin transporter and Na(+) ion release. *PLoS Comput Biol*. **7** (10): e1002246.

19. Koldso, H., Autzen, H. E., Grouleff, J. and Schiott, B. 2013. Ligand induced conformational changes of the human serotonin transporter revealed by molecular dynamics simulations. *PLoS One*. **8** (6): e63635.

20. Celik, L., Schiott, B. and Tajkhorshid, E. 2008. Substrate binding and formation of an occluded state in the leucine transporter. *Biophys J*. **94** (5): 1600-1612.

21. Lemmon, M. A., Treutlein, H. R., Adams, P. D., Brunger, A. T. and Engelman, D. M. 1994. A dimerization motif for transmembrane alpha-helices. *Nat Struct Biol*. **1** (3): 157-163.

22. Jess, U., Betz, H. and Schloss, P. 1996. The membrane-bound rat serotonin transporter, SERT1, is an oligomeric protein. *FEBS Lett*. **394** (1): 44-46.

23. Stockner, T., Jurik, A., Weissensteiner, R., Freissmuth, M., Ecker, G. F. and Sitte, H. H. 2014. Membrane Transport Mechanism. Springer. 99-120

24. Coleman, J. A., Green, E. M. and Gouaux, E. 2016. X-ray structures and mechanism of the human serotonin transporter. *Nature*. **532** (7599): 334-339.

25. de Jong, D. H., Singh, G., Bennett, W. D., Arnarez, C., Wassenaar, T. A., Schafer, L. V., Periole, X., Tieleman, D. P. and Marrink, S. J. 2012. Improved parameters for the martini coarse-grained protein force field. *J Chem Theory Comput*. **9** (1): 687-697.

26. Abagyan, R., Totrov, M. and Kuznetsov, D. 1994. ICM—a new method for protein modeling and design: applications to docking and structure prediction from the distorted native conformation. *J Comput Chem*. **15** (5): 488-506.

27. Ferraro, M., Masetti, M., Recanatini, M., Cavalli, A. and Bottegoni, G. 2015. Modeling lipid raft domains containing a mono-unsaturated phosphatidylethanolamine species. *RSC Adv*. **5** (47): 37102-37111.

28. Bond, P. J. and Sansom, M. S. 2006. Insertion and assembly of membrane proteins via simulation. *J Am Chem Soc*. **128** (8): 2697-2704.

29. Marrink, S. J., Risselada, H. J., Yefimov, S., Tieleman, D. P. and de Vries, A. H. 2007. The MARTINI Force Field: Coarse Grained Model for Biomolecular Simulations. *J Phys Chem B*. **111** (27): 7812-7824.

30. Berendsen, H. J., Postma, J. P. M., van Gunsteren, W. F., DiNola, A. and Haak, J. 1984. Molecular dynamics with coupling to an external bath. *J Chem Phys*. **81** (8): 3684-3690.

31. D'agostino, R. B. 1971. An omnibus test of normality for moderate and large size samples. *Biometrika*. **58** (2): 341-348.

32. D’Agostino, R. B. and Pearson, E. S. 1973. Testing for departures from normality. *Biometrika*. **60** 613-622.

33. Krzywinski, M. and Altman, N. 2013. Points of significance: Significance, P values and t-tests. *Nat Meth*. **10** (11): 1041-1042.

34. Whitley, E. and Ball, J. 2002. Statistics review 6: Nonparametric methods. *Crit Care*. **6** (6): 509-513.

35. Reimann, C., Filzmoser, P. and Garrett, R. G. 2005. Background and threshold: critical comparison of methods of determination. *Sci Total Environ*. **346** (1–3): 1-16.

36. Bewick, V., Cheek, L. and Ball, J. 2004. Statistics review 10: further nonparametric methods. *Crit Care*. **8** (3): 196-200.

37. Wilcoxon, F. 1945. Individual comparisons by ranking methods. *Biometrics bulletin*. 80-83.

38. Brasseur, R., Pillot, T., Lins, L., Vandekerckhove, J. and Rosseneu, M. 1997. Peptides in membranes: tipping the balance of membrane stability. *Trends Biochem Sci*. **22** (5): 167-171.

39. Lins, L. and Brasseur, R. 2008. Tilted peptides: a structural motif involved in protein membrane insertion? *J Pept Sci*. **14** (4): 416-422.

40. Fantini, J., Carlus, D. and Yahi, N. 2011. The fusogenic tilted peptide (67-78) of alpha-synuclein is a cholesterol binding domain. *Biochim Biophys Acta*. **1808** (10): 2343-2351.

41. Krishnamurthy, H. and Gouaux, E. 2012. X-ray structures of LeuT in substrate-free outward-open and apo inward-open states. *Nature*. **481** (7382): 469-474.

42. Fantini, J. and Barrantes, F. J. 2013. How cholesterol interacts with membrane proteins: an exploration of cholesterol-binding sites including CRAC, CARC, and tilted domains. *Front Physiol*. **4** 31.

43. Chattopadhyay, A. 2014. GPCRs: Lipid-Dependent Membrane Receptors That Act as Drug Targets. *Adv Biol*. **2014** 12.

44. Prasanna, X., Chattopadhyay, A. and Sengupta, D. 2014. Cholesterol modulates the dimer interface of the β 2-Adrenergic receptor via cholesterol occupancy sites. *Biophys J*. **106** (6): 1290-1300.

45. Sengupta, D. and Chattopadhyay, A. 2015. Molecular dynamics simulations of GPCR-cholesterol interaction: An emerging paradigm. *Biochim Biophys Acta*. **1848** (9): 1775-1782.
